# The MAP kinase ERK and its scaffold protein MP1 interact with the chromatin regulator Corto during *Drosophila *wing tissue development

**DOI:** 10.1186/1471-213X-11-17

**Published:** 2011-03-14

**Authors:** Emmanuèle Mouchel-Vielh, Julien Rougeot, Martine Decoville, Frédérique Peronnet

**Affiliations:** 1 Université Pierre et Marie Curie-Paris 6; Centre National de la Recherche Scientifique; UMR7622, Laboratoire de Biologie du Développement, Equipe Chromatine et Développement, 75005 Paris, France; 2 Centre de Biophysique Moléculaire, CNRS UPR 4301, conventionnée avec l'Université d'Orléans, 45071 Orléans, France

## Abstract

**Background:**

Mitogen-activated protein kinase (MAPK) cascades (p38, JNK, ERK pathways) are involved in cell fate acquisition during development. These kinase modules are associated with scaffold proteins that control their activity. In *Drosophila*, *dMP1*, that encodes an ERK scaffold protein, regulates ERK signaling during wing development and contributes to intervein and vein cell differentiation. Functional relationships during wing development between a chromatin regulator, the Enhancer of Trithorax and Polycomb Corto, ERK and its scaffold protein dMP1, are examined here.

**Results:**

Genetic interactions show that *corto *and *dMP1 *act together to antagonize *rolled *(which encodes ERK) in the future intervein cells, thus promoting intervein fate. Although Corto, ERK and dMP1 are present in both cytoplasmic and nucleus compartments, they interact exclusively in nucleus extracts. Furthermore, Corto, ERK and dMP1 co-localize on several sites on polytene chromosomes, suggesting that they regulate gene expression directly on chromatin. Finally, Corto is phosphorylated. Interestingly, its phosphorylation pattern differs between cytoplasm and nucleus and changes upon ERK activation.

**Conclusions:**

Our data therefore suggest that the Enhancer of Trithorax and Polycomb Corto could participate in regulating vein and intervein genes during wing tissue development in response to ERK signaling.

## Background

The mitogen-activated protein kinase (MAPK) pathways are evolutionary conserved signaling pathways used by eukaryotic cells to regulate gene expression during diverse processes such as proliferation, differentiation, apoptosis, adaptation to changes in their environment, and so on (for a review, see [1]). MAPK proteins are serine-threonine kinases that can phoshorylate targets in the cytoplasm or the nucleus in response to stimuli such as mitogenic or stress signals. MAPKs can be grouped into three classes depending on the stimuli they respond to. Extracellular regulated kinases (ERK) are mainly activated by mitogenic stimuli such as growth factors and hormones, whereas c-Jun N-terminal kinases (JNK) and p38 kinases respond predominantly to stress stimuli. Kinases associate with scaffold proteins that regulate signaling by providing critical spatial and temporal specificities. Notably, the scaffold protein MP1 forms a signaling complex with MEK and ERK thus facilitating ERK activation [2,3]. One of the best characterized mechanisms by which MAPKs regulate gene expression involves phosphorylation of transcription factors, which consequently modifies their activity, regulating either their intracellular location, their stability, their binding to DNA, or their interactions with regulatory proteins (for a review, see [4]).

Although the traditional view has been that most phosphorylation events do not occur directly at promoters of genes that are ultimately controlled by MAPK pathways, recent reports have highlighted some cases where MAPKs are integral components of transcriptional activation complexes. For example, during mammalian myoblast differentiation, p38 is recruited to the promoters of myogenic genes together with the muscle-regulatory factors MyoD and MEF2C [5]. In pancreatic β-cells, in response to increased glucose concentration, ERK1/2 MAPKs are bound to the insulin gene promoter in the same complex as their transcription factor substrates MafA, Beta2 and PDX-1 [6]. In yeast, the p38-related Hog1 kinase coordinates the transcriptional program required for cell survival upon osmostress: active Hog1 interacts with the transcription factor Hot1, inducing recruitment of Hog1 to osmostress-responsive promoters [7]. Anchoring of Hog1 to chromatin was shown to be important to stimulate the recruitment and activation of RNA Pol II [8]. In addition to this role in transcriptional initiation, Hog1 also behaves as a transcriptional elongation factor [9]. Genome-wide chromatin immunoprecipitation coupled with microarrays (ChIP-Chip) experiments have revealed that two other yeast MAPKs involved in pheromone response, Fus3 and Kss1, are bound to several genes that are expressed upon pheromone pathway activation [10].

Once bound to chromatin, MAPKs do not only modulate transcription factor activity and RNA Pol II recruitment, but also regulate gene expression by inducing changes in chromatin organization and epigenetic histone modifications. Indeed, yeast Hog1 facilitates recruitment of either the histone deacetylase Rpd3-Sin3 complex [11], the SAGA complex which contains both histone acetylation and de-ubiquitylation activities [12], or the SWI-SNF chromatin remodeling complex [13]. During mammalian myoblast differentiation, p38 targets the SWI-SNF complex [5] as well as the ASH2L Trithorax complex, that contains a histone methyl-transferase, to muscle-specific genes [14]. It is tempting to speculate that binding of these complexes to chromatin relies on phosphorylation of some of their components by MAPKs. For example in mammals, the downstream MAPKAP3 kinase, once activated by phosphorylation in response to mitogenic or stress signals, phosphorylates some members of the chromatin Polycomb Repressive Complex 1 (PRC1). This results in dissociation of PRC1 from chromatin and subsequent de-repression of target genes [15]. Altogether, these data show that chromatin reorganization mediated by nucleosome remodeling and epigenetic mark modifications is an important process to regulate gene expression in response to MAPK signaling. This process could involve a dynamic switch between the binding of either a silencing complex or an activating complex on a target gene.

In most of the examples mentioned above, these repressing and activating complexes are formed by proteins of the Polycomb-group (PcG) and Trithorax-group (TrxG) which combine into several heteromeric complexes that bind chromatin. PcG and TrxG complexes regulate gene expression by modulating chromatin structure, in particular by depositing specific post-translational histone modifications and by nucleosome remodeling. PcG complexes lead to compact, transcriptionally inactive chromatin, whereas TrxG complexes counteract PcG-mediated repression and maintain chromatin in an open conformation that facilitates transcription (for a review, see [16]). A third class of proteins, the Enhancers of Trithorax and Polycomb (ETP), is involved in PcG- as well as TrxG-mediated gene regulation (for a review, see [17]). Interestingly, ETPs allow the recruitment on chromatin of either PcG or TrxG complexes and could therefore participate in a switch between activation and repression. The first *ETP *mutants have been identified in *Drosophila *as enhancers of both *Polycomb-group (PcG) *and *trithorax-group (trxG) *mutations [18]. Although not found in any of the PcG or TrxG core complexes purified so far, ETPs interact with these complexes and are required for both PcG-mediated silencing and TrxG-mediated activation. For example, mutants of the ETP gene *Asx *enhance homeotic phenotypes of both *PcG *and *trxG *mutations [19]. The GAGA factor encoded by *Trithorax-like *(*Trl*) was first described as an activator of *Hox *gene expression and later shown to play a role in recruitment of PcG complexes [20,21]. The HMGB protein DSP1 behaves as an ETP since a *dsp1 *null allele exhibits a *PcG *phenotype but enhances at the same time the phenotype of several *trxG *mutants [22]. Furthermore, DSP1 is required for PcG complex recruitment to chromatin [23]. Lastly, *corto*, which is ubiquitously expressed all along development, presents the characteristics of an *ETP *since loss-of-function mutants exhibit both *PcG *and *trxG *phenotypes and enhance the phenotype of some *PcG *as well as *trxG *mutants [24,25]. The Corto protein interacts not only with PcG and TrxG proteins, but also with other ETPs such as GAGA and DSP1, which suggests that different combinations of ETPs could favor the recruitment of either PcG or TrxG complexes on chromatin [26,27]. Nevertheless, the mechanism through which ETPs could exert this dual function remains to be investigated.

*corto *loss-of-function mutants exhibit several phenotypes, among them ectopic veins on wings which recall the phenotype induced by a gain-of-function mutation of *rolled (rl) *that encodes the MAPK ERK [25,28]. In *Drosophila*, specification and differentiation of wing tissues (*i.e*. vein and intervein) occur in wing imaginal discs during the third larval and pupal stages and rely on several developmental signals including those mediated by EGF, BMP, Hedgehog and Wnt (for a review, see [29]). Signaling mediated by the *Drosophila *EGF Receptor (DER) is crucial for early specification of the longitudinal vein primordia called proveins, as well as for differentiation of vein and intervein cells (for a review, see [30]). Once activated by one of its ligands, DER activates a phosphorylation cascade leading to ERK signaling. Early ERK signaling in wing discs of third instar larvae specifies provein [31]. In provein territories, ERK maintains expression of *rhomboïd *(*rho*), which is required to direct provein cells to differentiate as vein cells [32]. *rho *encodes a serine-threonine protease which is required to process EGFR ligands and thus participates in a positive feed-back loop that maintains high levels of ERK activity [33,34]. On the other hand, ERK signaling represses *blistered *(*bs*) expression. *bs*, that encodes a homolog of the mammalian Serum Response Factor (SRF), is expressed in the future intervein cells and controls the specification of intervein tissue [35,36]. Later during development, at the pupal stage, ERK signaling is also required to promote intervein cell differentiation [31]. The formation of vein and intervein tissues thus depends on the outcome of a fine-tuned balance between *rho *and *bs *expression patterns, which are both regulated by ERK signaling. Furthermore, the scaffold protein dMP1 also participates in ERK signaling during vein and intervein differentiation [3]. The wing phenotype of *corto *mutants, but also the fact that we isolated dMP1 in a two-hybrid screen using Corto as bait, prompted us to address the potential role of this ETP in relation to ERK signaling during wing vein and intervein differentiation. Our genetic interactions between *corto *and genes encoding some actors of the ERK signaling pathway, *i.e. rl *itself, *dMP1, bs *and *rho *show that *corto *and *dMP1 *contribute to antagonize *rl *vein-promoting function in future intervein cells. Biochemical analyzes show that Corto interacts directly with ERK. Furthermore, Corto is phosphorylated and its phosphorylation increases upon ERK activation. Surprisingly, ERK and dMP1 associate with Corto exclusively in the nucleus. As suggested by immunolocalizations on polytene chromosomes, a dMP1/ERK/Corto complex might be targeted to chromatin to directly regulate gene expression, thus allowing proper wing tissue differentiation.

## Results and Discussion

### *corto *contributes to intervein tissue differentiation

**Figure 1:**
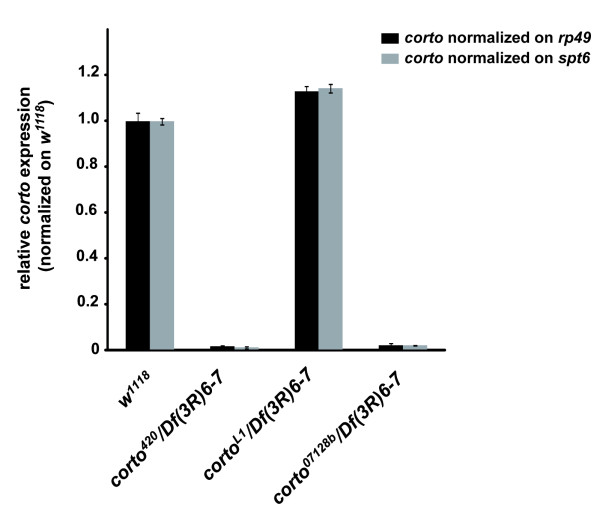
**Quantification of *corto *transcripts by quantitative RT-PCR on wing discs from *corto^420^/Df(3R)6-7*, *corto^L1^/Df(3R)6-7 *and *corto^07128b^/Df(3R)6-7 *third instar larvae**. The relative *corto *expression level was obtained by normalization on *rp49 *(black bars) or *spt6 *(grey bars) level. For each mutant genotype, this normalized level was compared to that of wild type (*w^1118^*) wing discs.

We first investigated the ectopic vein phenotype of *corto *mutants using three different recessive lethal alleles: *corto^420^*, *corto^07128b ^*and *corto^L1^*. *corto^420 ^*is a deletion of the *corto *locus [25], corto*^07128b ^*a P-element insertion located 0.5 kb upstream of *corto *5'-UTR [37], and corto*^L1 ^*an EMS-induced mutation [38]. As already described for *corto^420 ^*[24,25], heteroallelic combinations using *corto^07128b^*, *corto^L1 ^*and a deficiency encompassing *corto *[*Df(3R)6-7*] are poorly viable, since we observed 0% to 10% escapers depending on combinations. Therefore, these three alleles are true loss-of-function alleles. This was confirmed by quantitative RT-PCR analysis on wing discs from third instar larvae, that showed absence of *corto *transcripts in *corto^420^*/*Df(3R)6-7 *and *corto^07128b^*/*Df(3R)6-7 *larvae (Figure 1). In contrast, *corto^L1^/Df(3R)6-7 *larvae exhibited the same level of *corto *transcripts as wild-type flies, which suggests that the mutation in *corto^L1 ^*rather affects the level or activity of Corto protein.

**Figure 2:**
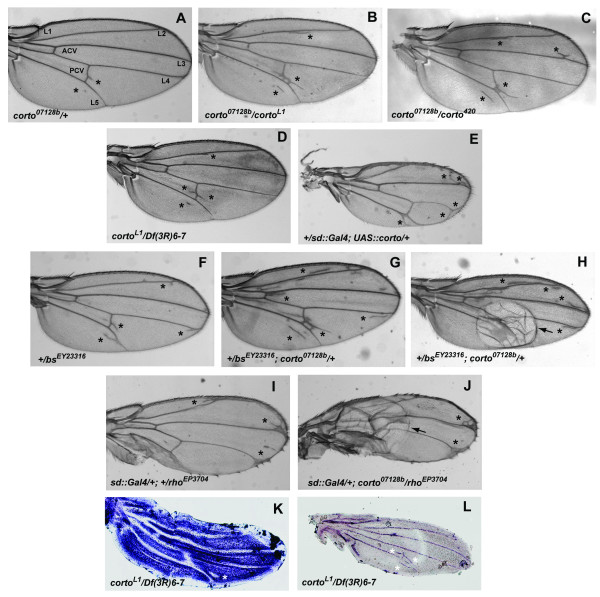
***corto *regulates wing tissue differentiation in interaction with *blistered *(*bs) *and *rhomboïd *(*rho*)**. (A): Wings from *corto^07128b^/+ *flies exhibit ectopic veins (shown by asterisks) within intervein tissue (L1-L5: longitudinal veins; ACV and PCV: anterior and posterior cross-veins). (B, C, D): This phenotype is more severe in flies heteroallelic for *corto *loss-of-function mutations (*corto^07128b^/corto^L1^; corto^07128b^/corto^420^; corto ^L1^/Df(3R)6-7 *). (E): *corto *over-expression induces ectopic veins (shown by asterisks). (F to J): *corto^07128b ^*enhances the ectopic vein phenotype (shown by asterisks) and the blistered phenotype (shown by arrows) induced by the *bs^EY23316 ^*loss-of-function allele (F, G, H) and by *rho *over-expression using the *rho^EP3704 ^*allele (I, J). (K, L): *in situ *hybridization of 30 h pupal wings from *corto^L1^/Df(3R)6-7 *mutants with *bs *probe (K) or *rho *probe (L); white asterisks point to *bs *and *rho *deregulation.

As already described [25], *corto^420^/+ *heterozygous flies exhibited very few ectopic veins (2.2% to 8.6%). This phenotype was more penetrant in *corto^L1^/+ *(49% to 52.2%) and *corto^07128b^/+ *(97.4% to 97.8%) heterozygous flies (Table 1 and Figure 2A). Since both *corto^420 ^*and *corto^07128b ^*are devoid of *corto *transcripts, the discrepancy between these alleles may be a consequence of an interaction with the genetic background. For all combinations, the few heteroallelic *corto *escapers displayed a stronger ectopic vein phenotype than *corto *heterozygous flies (Figure 2B-D). Ectopic veins mainly arose close to longitudinal veins 2, 3, 5 and to the posterior cross-vein, which seems to be the case for most mutations that induce ectopic vein phenotypes [32]. Interestingly, over-expressing *corto *using a *UAS::corto *construct and the wing specific *Beadex::Gal4 *(*Bx::Gal4*, data not shown) or *scalloped::Gal4 *(*sd::Gal4*) driver also induced extra pieces of vein tissue in all flies (Figure 2E). Since both *corto *over-expression and loss-of-function induced the same phenotype, one possibility is that Corto may be required in stoechiometric amount to allow correct wing tissue differentiation. This feature characterizes proteins that act through formation of complexes. Indeed, complexes are very sensitive to the relative amounts of their components, and can be disrupted either by an excess or a shortage of one of these [39].

**Table 1:** *corto *mutants induce ectopic vein phenotypes and interact with *blistered (bs) *and *rhomboïd (rho) *during wing tissue differentiation

Genotype	Total females observed	% females with ectopic veins only	% females with blistered wings
*corto^420^/+*	70	8.6	0
*+/corto^420^*	45	2.2	0
*corto^L1^/+*	51	49	0
*+/corto^L1^*	90	52.2	0
*corto^07128b^/+*	78	97.4	0
*+/corto^07128^*	46	97.8	0
			
+/*bs^EY23316^*	136	100	0
+/*bs^EY23316 ^*; *corto^07128b/+^*	80	67.5	32.5^a^
			
+/*rho^EP3704^*	68	0	0
*sd::Gal4/+; +/rho^EP3704^*	105	90.5	9.5
*sd::Gal4*/+; *corto^420 ^*/*rho^EP3704^*	34	58.8	41.2 ^a^
*sd::Gal4/+; corto^07128b ^/rho^EP3704^*	90	51.1	48.9 ^a^

For all genotypes, the first allele was brought by the mother. Only female phenotypes are reported here, but similar results were obtained for males. Numbers of females with blistered wings in flies containing a *corto *mutation and the *bs^EY23316 ^*or *rho^EP3704 ^*allele were compared to those of flies containing the *bs^EY23316 ^*or *rho^EP3704 ^*allele only (Z-test, ^a ^p < 0.001).

In order to assess the temporal requirement for *corto *function in wing tissue differentiation, we crossed the *UAS::corto *line with the *hs::Gal4 *driver strain allowing staged *Gal4 *expression (Table 2). The highest percentage of ectopic vein phenotype was obtained when heat-shock was applied between 96 to 120 hours after egg laying, which corresponds to the mid to late third instar larval stage. Interestingly, it has been shown that, from late third instar larval stage to pupal stage, down-regulation of ERK signaling is crucial for wing tissue formation: indeed, expression of a constitutively active form of the MAPKK Raf at the third instar larval stage induces vein loss, whereas expression of a dominant negative form of the receptor DER at pupal stage leads to formation of ectopic veins [31].

**Table 2:** Staged *corto *over-expression induced ectopic vein phenotypes mostly during late larval development

Genotype	*+/hs::Gal4*		*UAS::corto/hs::Gal4*	
**Time of heat-shock (hours After Egg Laying AEL)**	**Total females observed**	**% females with ectopic veins**	**Total females observed**	**% females with ectopic veins**

No heat-shock	32	0	195	17.9
48 h-72 h AEL (L2 larvae)	127	3.1	182	25.8
72 h-96 h AEL (early to mid L3 larvae)	123	3.2	235	29.8
96 h-120 h AEL (mid to late L3 larvae)	56	3	216	46.3^a^
120 h-136 h AEL (young pupae)	78	5.1	309	31.7

For all genotypes, the first allele was brought by the mother. Only female phenotypes are reported here, but similar results were obtained for males. The number of females with ectopic veins when heat-shock was applied between 96 h-120 h AEL was compared to the ones of females with ectopic veins obtained after heat-shock applied between 72 h-96 h AEL or 120 h-136 h AEL (Z-test, ^a ^p < 0.001).

In conclusion, *corto *misregulation (either loss-of-function or over-expression) induced ectopic veins that formed within intervein tissue and never truncated veins. This observation suggested that Corto contributes to intervein tissue differentiation, whereas it does not seem to be involved in vein formation. We have previously shown that *corto *interacts with some TrxG genes during wing tissue formation. Indeed, *moira*, *kismet *and *ash1 *mutants enhance the ectopic vein phenotype of *corto^420 ^*[25]. Furthermore, several *corto *alleles enhance the ectopic vein phenotype of mutations in *snr1 *that encodes a component of the SWI/SNF complex [38,40], a chromatin-remodeling complex also involved in wing tissue differentiation [41-43]. One hypothesis is that Corto, as an ETP, could participate in the recruitment of TrxG complexes to regulate expression of genes involved in wing tissue differentiation.

To clarify the role of *corto *in the formation of intervein tissue, we performed genetic interaction assays between *corto *and the intervein-promoting gene *blistered *(*bs*), or the vein-promoting gene *rhomboïd *(*rho*). As expected for a *bs *loss-of-function allele [35,36], wings of flies heterozygous for *bs^EY23316 ^*exhibited a moderate ectopic vein phenotype, but none showed blisters in the wings (Table 1 and Figure 2F). *corto^07128b ^*enhanced the ectopic vein phenotype induced by *bs^EY23316 ^*(compare Figure 2G to Figure 2F). In addition, 32.5% of these trans-heterozygous flies had blisters in the wings (Table 1 and Figure 2H). These blisters, which result from impaired adhesion between the ventral and dorsal wing surfaces, could be caused by formation of many vein cells within intervein tissue. They are frequently observed in *bs *mutants [35] or when *rho *is over-expressed [36]. This result therefore showed that *bs *and *corto *act synergistically to promote intervein cell fate. Ectopic over-expression of *rho *using the *rho^EP3704 ^*allele and the *sd::Gal4 *driver induced ectopic veins for most of the flies and in a few cases (9.5%) formation of blisters (Figure 2I and Table 1). This phenotype was similar to that induced by over-expressing *rho *under control of a heat-inducible promoter [32]. Both *corto^420 ^*and *corto^07128b ^*alleles enhanced this phenotype since the number of flies with blisters in the wings significantly increased (Table 1 and Figure 2J). This observation showed that *corto *antagonizes *rho *in vein formation.

Taken together, these results suggest that *corto *might antagonize *rl *vein-promoting function in future intervein cells. *corto *misregulation could therefore lead to deregulation of certain vein and intervein-promoting genes. Indeed, we observed deregulation of *bs *and *rho *in some intervein cells of pupal wings from *corto^L1^/Df(3R)6-7 *escapers: in these cells, *bs *is down-regulated (Figure 2K) whereas *rho *is ectopically expressed (Figure 2L).These cells could thus acquire a vein fate.

### ***Corto *and *dMP1 *act together and participate in the control of wing tissue differentiation**

**Figure 3:**
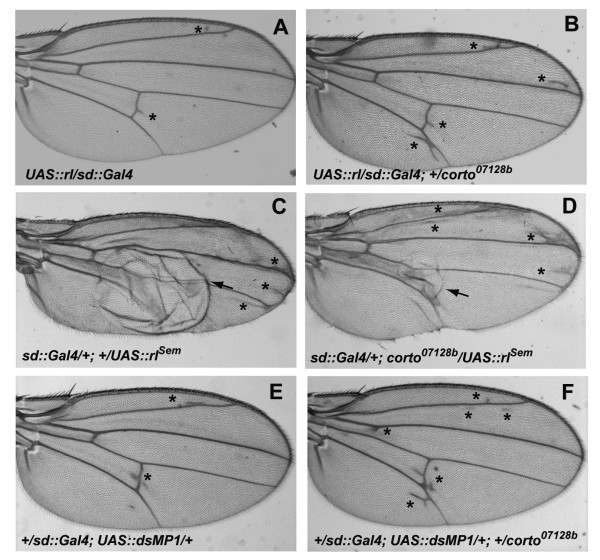
***corto *interacts with *rl *and *dMP1 *during wing development**. (A, B): *corto *loss-of-function (*corto^07128b^*) enhances the ectopic vein phenotype (shown by asterisks) induced by *rl *over-expression. (C, D): *corto *loss-of-function (*corto^07128b^*) diminishes the blistered phenotype (shown by arrows) induced by *rl^Sem ^*over-expression. (E, F): *corto *loss-of-function (*corto^07128b^*) enhances the ectopic vein phenotype (shown by asterisks) induced by *dMP1 *down-regulation.

Since the wing phenotype of *corto *mutants resembles the one induced by hyperactivation of ERK signaling pathway, we wondered whether *corto *was involved in the regulation of this pathway during wing development. We first tested genetic interactions between *corto *and *rolled *using the *UAS::rolled *strain which allows targeted ERK over-expression when crossed with a *Gal4 *driver. All flies over-expressing *rolled *with the *sd::Gal4 *driver at 25°C exhibited a mild ectopic vein phenotype ([3] and Figure 3A). Expressivity of this phenotype was enhanced by the *corto^07128b ^*allele (Figure 3B). This result suggests that the roles of *corto *and *rolled *in vein-promoting function are antagonistic.

We also used the *UAS::rolled^Sem ^*(*rl^Sem^*) transgene that encodes a hyper-active form of ERK [28,44]. At 18°C, flies that over-expressed the *UAS::rl^Sem ^*transgene under control of the *sd::Gal4 *driver exhibited ectopic veins. This phenotype was much stronger than the one induced by *rolled *over-expression (compare Figure 3 to 3). For 88% of these flies, this phenotype was very strong since one or the two wings showed blisters (Table 3 and Figure 3C). Surprisingly, penetrance and expressivity of the *rl^Sem ^*over-expression phenotype were lowered by *corto^420 ^*and *corto^07128b ^*alleles, as only 49.4% of *corto^420 ^*flies and 55.1% of *corto^07128b ^*ones exhibited blisters in one or both wings (Table 3) and blisters were smaller (Figure 3D). This result confirmed that *corto *and *rl *interact during wing tissue formation. However, the observation that *corto *mutation enhanced a mild-activation of ERK pathway (as induced by *UAS::rl*) whereas slowing-down a hyper-activation (as induced by *UAS::rl^Sem^*) is paradoxical and requires further experiments to be fully understood.

**Table 3:** *corto *interacts with *rolled (rl) *and *dMP1 *during wing tissue differentiation

Genotype	Total females observed	% females with ectopic veins only	% females with one blistered wing	% females with two blistered wings
*sd::Gal4/+;+/UAS::rl^Sem^*	108	12	40.7	47.3
*sd::Gal4/+;corto^420^/UAS::rl^Sem^*	85	50.6 ^a^	36.5	12.9 ^a^
*sd::Gal4/+;corto ^07128b^/UAS::rl^Sem^*	78	44.9 ^a^	44.9	10.2 ^a^
				
*+/sd::Gal4; UAS::dsMP1/+*	158	78.5	0	0
*+/sd::Gal4; UAS::dsMP1/+; +/corto^420^*	90	92.2 ^b^	0	0
*+/sd::Gal4; UAS::dsMP1/+; +/corto^07128b^*	92	100	0	0

For all genotypes, the first allele was brought by the mother. Only female phenotypes are reported here, but similar results were obtained for males. Numbers of females with ectopic veins only or with two blistered wings among flies bearing a *corto *allele and over-expressing *rl^Sem ^*were compared to the one of flies over-expressing *rl^Sem ^*only. Numbers of females with ectopic veins among flies containing the *UAS::dsMP1 *transgene, the *sd::Gal4 *transgene and a *corto *mutation were compared to that of flies containing the *UAS::dsMP1 *and the *sd::Gal4 *transgenes only (Z-test, ^a ^p < 0.001; ^b ^p < 0.05).

**Figure 4:**
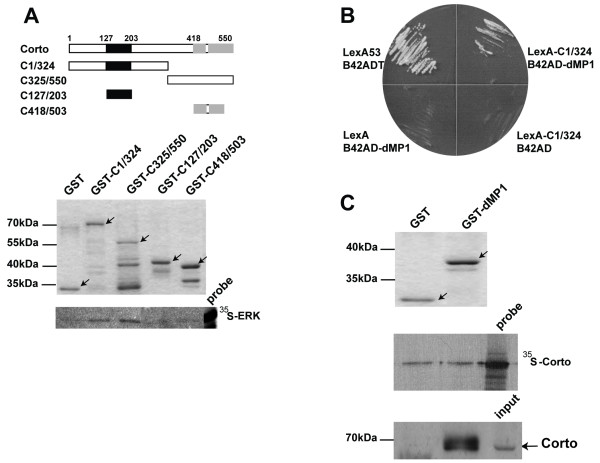
**Corto interacts *in vitro *directly with ERK and indirectly with dMP1**. (A): GST pull-down assays show direct interaction between Corto and ERK. Top: Schematic representation of Corto full-length and truncated forms (black box: chromodomain; grey boxes: COOH-terminal globular domains). Bottom: Coomassie staining of fusion proteins (shown by arrows) and autoradiography of the GST pull-down assays with *in vitro *translated ^35^S-ERK. (B): Leucine test of two-hybrid experiment shows that the NH_2_-terminal half of Corto (LexA-C1/324 corresponding to amino-acids 1-324) interacts with full length dMP1 (B42AD-dMP1). Negative controls: LexA and B42AD-dMP1; LexA-C1/324 and B42AD; positive controls: LexA53 and B42ADT. (C): GST pull-down assays using a GST-dMP1 fusion protein show that the Corto-dMP1 interaction is not direct. Top: Coomassie staining of fusion proteins (shown by arrows). Middle: autoradiography of the GST pull-down assays showing no specific interaction between GST-dMP1 beads and *in vitro *translated ^35^S-Corto. Bottom: peptide pull-down assay using total embryonic protein extract and GST-dMP1 shows that Corto (revealed by anti-Corto antibodies) is specifically retained on GST-dMP1 beads (shown by an arrow).

We have recently shown that the *Drosophila *ortholog of *MP1*, *dMP1*, antagonizes *rl *vein-promoting function in the future intervein cells of the wing [3]. Furthermore, we isolated dMP1 in a two-hybrid screen using Corto as bait (see below and Figure 4B). We thus tested the genetic interactions between *corto *and *dMP1*. As already described [3], down-regulation of *dMP1 *by RNA interference using the *sd::Gal4 *driver induced ectopic veins in 78.5% of flies (Table 3 and Figure 3E). This percentage increased to 92.2% and 100% in combination with *corto^420 ^*or *corto^07128b^*, respectively (Table 3). With *corto^07128b^*, the expressivity of the ectopic vein phenotype was also enhanced (compare Figure 3F to Figure2A and 3E). Therefore, these results showed that *corto *and *dMP1 *act synergistically and participate in intervein tissue differentiation in response to ERK signaling.

### Corto interacts *in vitro *directly with ERK and indirectly with dMP1

We have previously shown that dMP1 forms a complex with ERK, which is required for the proper development of intervein cells [3]. To understand the molecular bases of the relationship between Corto, dMP1 and ERK, we first questioned the physical interaction between Corto and ERK. We carried out GST pull-down assays using *in vitro *translated ERK and GST-Corto fusion proteins. Structural analysis of Corto has shown that this 550 amino-acid protein contains three globular domains that might correspond to functional domains ([26] and Figure 4A). The first one is located at position 127-203 and exhibits strong structural similarities with chromodomains, that are chromatin targeting modules found in some regulators of chromatin structure (for a review, see [45]). The two others, located at positions 418-455 and 480-550, present no obvious similarities with known protein domains. *In vitro *translated ERK protein was retained on GST-C1/324 and GST-C325/550 beads containing the NH_2_-terminal half and the COOH-terminal half of Corto, respectively (Figure 4A). In contrast, ERK was not retained on GST-C127/207 beads containing the Corto chromodomain, or on GST-C418/503 beads containing part of the two COOH-terminal globular domains. The lack of interaction with GST-C127/207 and GST-C418/503 suggested that none of these domains was sufficient to mediate Corto-ERK interaction, either because of inappropriate folding of these short domains in the GST fusion proteins, or because none of these two fragments contains the sequences that mediate ERK binding. Taken together, these results showed that Corto interacts directly with ERK *in vitro*. Further experiments are needed to determine the precise domains or residues that mediate the interaction between Corto and ERK.

Since we isolated dMP1 in a two-hybrid screen using the NH_2_-terminal part of Corto as a bait (Figure 4B), we next questioned the physical interaction between Corto and dMP1. We performed GST pull-down assays using GST-dMP1 fusion protein and *in vitro *translated Corto to see whether their interaction was direct or indirect. Indeed, indirect interactions *via *yeast proteins have already been observed in two-hybrid experiments [46]. As shown in the middle panel of Figure 4C, the same result was obtained using GST or GST-dMP1 beads indicating that there was no specific direct interaction between Corto and dMP1. However, by incubating GST-dMP1 beads with total embryonic protein extract, we observed after blotting with anti-Corto antibodies that Corto was specifically retained on GST-dMP1 beads (bottom panel of Figure 4C). Therefore, we concluded that Corto and dMP1 interact *via *additional factors. One potential candidate could be ERK, since it directly interacts with Corto (as shown above) and with dMP1 [3].

### Corto is located both in the cytoplasm and the nucleus and is phosphorylated

**Figure 5:**
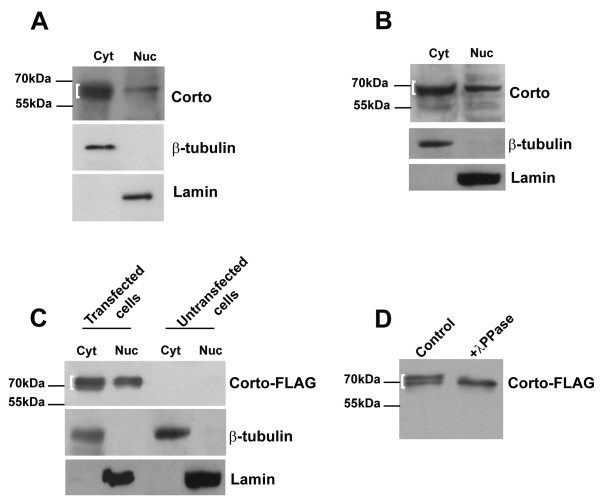
**Corto is a phosphorylated protein located both in the cytoplasm and in the nucleus**. (A to C): Corto is present in the cytoplasm and in the nucleus and exhibits several isoforms very close in size (indicated by a brace). In the nucleus, the higher isoforms are predominant. (A, B): Western-blot using anti-Corto antibodies and cytoplasmic or nuclear extracts from wild-type embryos (A) or S2 cells (B). (C): Western-blot with anti-FLAG antibodies using cytoplasmic and nuclear extracts from S2 cells expressing or not a Corto-FLAG fusion protein. Cyt: 50 μg cytoplasmic extract; Nuc: 10 μg nuclear extract. In A, B, C, β-tubulin and lamin were used as cytoplasmic and nuclear loading controls, respectively. (D): The different Corto isoforms correspond to proteins that are differently phosphorylated: Western-blot with anti-FLAG antibodies after phosphatase treatment of 15 μg total extract from S2 cells expressing a Corto-FLAG fusion protein shows that the upper band disappears. In this experiment, only the two major Corto-FLAG isoforms are visible because of the low amount of total extract loaded.

Upon activation of the MAPK cascade, ERK is phosphorylated in the cytoplasm. Di-phosphorylated ERK (dP-ERK) phosphorylates in turn a large number of targets with diverse functions and different subcellular localizations. In particular, part of dP-ERK is translocated into the nucleus where it phosphorylates some transcription factors (for reviews, see [1,4]). Mammalian MP1 is present in the cytoplasm in association with ERK but its possible nuclear localization has not been reported [47,48]. Nevertheless, we have recently shown that dMP1 is present both in the cytoplasm and the nucleus [3]. We thus asked whether Corto was present in the same compartments as ERK and dMP1. Corto was detected in nuclear and cytoplasmic extracts from embryos (Figure 5A), third instar larvae (data not shown) and Schneider S2 cells (Figure 5B). A similar nuclear and cytoplasmic distribution was observed for a Corto-FLAG fusion protein expressed in S2 cells (Figure 5C). Both Corto and Corto-FLAG exhibited several isoforms very close to each other in size, with the lowest isoforms being more abundant in the cytoplasm than in the nucleus (Figure 5A-C). Corto is very rich in serine (16%), threonine (4.5%), tyrosine (2.9%), and presents many predicted phosphorylation sites for several kinases distributed all along the sequence (according to NetPhos predictions: 28 phosphorylable serines, 6 threonines, and 3 tyrosines). Hence we checked whether the isoforms we observed could indeed correspond to differentially phosphorylated molecules. As shown in Figure 5D, the upper bands of Corto-FLAG disappeared after lambda phosphatase treatment. Altogether, these results showed that Corto is a phosphorylated protein present both in the cytoplasm and in the nucleus. In addition, the Corto phosphorylation pattern seemed to be different between the two cellular compartments, with enrichment in highly phosphorylated forms in the nucleus. Phosphorylation of M33, a chromatin regulator, has been shown to regulate its nuclear translocation [49]. Further experiments are required to determine if Corto localization is regulated through a similar mechanism.

### The phosphorylation pattern of Corto is controlled at least partially by ERK pathway

**Figure 6:**
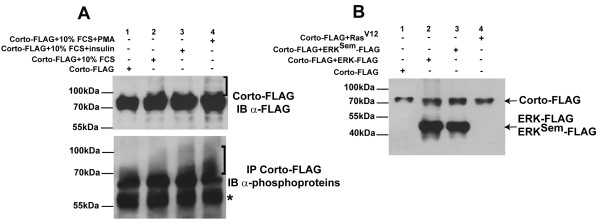
**The phosphorylation pattern of Corto depends on the level of ERK pathway activity**. (A): S2 cells in 0% fetal calf serum were transfected with a plasmid allowing Corto-FLAG over-expression. 48 hours after transfection, they were submitted to short ERK pathway activation by a 15 minutes treatment with either 10% fetal calf serum (lane 2), 10% fetal calf serum plus insulin 10 μg/ml (lane 3) or 10% fetal calf serum plus PMA 100 ng/ml (lane 4). Total protein extracts were either directly analyzed by Western blot with anti-FLAG antibodies (upper panel), or submitted to immunoprecipitation using anti-FLAG antibodies followed by immunoblotting with anti-phosphoprotein antibodies (lower panel). The braces point to the upper Corto isoforms that appear in response to ERK activation. The asterisk on the lower panel shows the heavy IgG chains, which have already been shown to be phosphorylated [62]. (B): Serum-starved S2 cells were either transfected with a plasmid allowing Corto-FLAG expression (lane 1), or co-transfected with Corto-FLAG and ERK-FLAG (lane 2), Corto-FLAG and ERK^Sem^-FLAG (lane 3) or Corto-FLAG and Ras^V12 ^(lane 4), in order to constitutively activate the ERK pathway. After 48 hours, total protein extracts were analyzed by Western blot using anti-FLAG antibodies.

Since Corto is phosphorylated and interacts *in vitro *with ERK, it is tempting to speculate that Corto could be phosphorylated in response to activation of the corresponding MAP kinase cascade. To answer this question, we transfected S2 cells in absence of serum with a plasmid allowing Corto-FLAG expression. After two days, we transiently activated the MAP kinase cascade by a short (15 minutes) treatment either with serum, with serum plus insulin or with serum plus PMA. In all three conditions, faint upper Corto isoforms appeared (brace on Figure 6A, upper panel), showing that Corto phosphorylation was induced very rapidly upon ERK activation. To confirm the existence of these faint upper isoforms, we immunoprecipitated Corto from these extracts using anti-FLAG antibodies and blotted the immunoprecipitates with antibodies directed against phosphoproteins (Figure 6A, lower panel). This experiment revealed first that Corto is constitutively phosphorylated even without MAP kinase activation. Second, ERK activation induced an hyperphosphorylation of Corto since an upper smear containing several bands very close in size appeared in all three conditions of activation tested.

As another way to activate the ERK pathway, we co-expressed Corto-FLAG and tagged forms of either ERK, ERK^Sem ^or Ras^V12 ^in S2 cells. Similar to ERK and ERK^Sem ^over-expression, over-expression in flies of a constitutively active form of Ras, Ras^V12^, induces ectopic vein cells [50]. Surprisingly, smaller Corto isoforms appeared when constitutively activating the ERK pathway with either ERK, ERK^Sem ^or Ras^V12 ^(Figure 6B). One possibility is that these smaller isoforms could correspond to partially dephosphorylated Corto molecules. Taken together, these experiments demonstrate that Corto presents a complex phosphorylation pattern that depends at least on ERK signaling. It is tempting to speculate that some phosphorylations are performed directly by ERK. Indeed, Corto contains 3 SP sites at positions 139, 190 and 428 that correlate with theoretical ERK1/ERK2 phosphorylation sites [51]. Identification of Corto phosphorylation sites as well as phospho-mutant analysis and determination of Corto phosphorylation status when bound to chromatin would help to better understand the role of these phosphorylation events.

### Interaction between ERK and dMP1 or Corto takes place in the nucleus only

**Figure 7:**
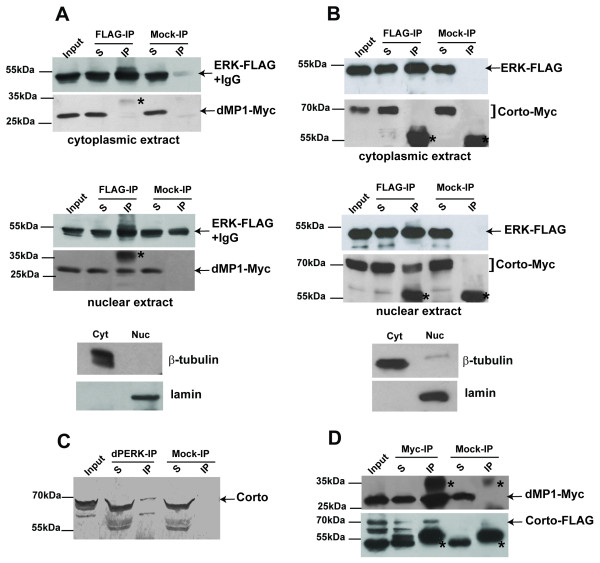
**Corto interacts *in vivo *with ERK in the nucleus only**. (A, B): ERK-FLAG co-immunoprecipitates with dMP1-Myc (A) and Corto-Myc (B) only in nuclear extracts. Immunoprecipitation was performed with either anti-FLAG (FLAG-IP) or anti-HA (Mock-IP) antibodies. Cytoplasmic (Cyt) and nuclear (Nuc) extracts from S2 cells expressing tagged proteins were analyzed by Western blot using β-tubulin and lamin as cytoplasmic and nuclear loading controls, respectively. Note that ERK-FLAG (about 50 kDa) co-migrates with the heavy IgG chains (asterisks). (C): Corto co-immunoprecipitates with the di-phosphorylated form of ERK (dP-ERK) in a total embryonic extract. Immunoprecipitated proteins were revealed using rat anti-Corto antibodies. (D): dMP1-Myc co-immunoprecipitates with Corto-FLAG only after cross-linking of cells before protein extraction. Immunoprecipitation was performed using either anti-Myc (Myc-IP) or anti-HA (Mock-IP) antibodies. In A, B, D, immunoprecipitated proteins were revealed by Western-blot using anti-FLAG or anti-Myc antibodies. Arrows and braces show immunoprecipitated tagged proteins and asterisks point to heavy or light IgG chains. In A and D, the light IgG chains in the mock-IP lanes are poorly recognized by the secondary antibodies, but are clearly visible when membranes were stained with Ponceau red (data not shown). S: supernatant after immunoprecipitation; IP: protein G-agarose beads. 5% of the input and 50% of the immunoprecipitate were loaded onto the gel.

We next performed co-immunoprecipitation experiments to see whether Corto interacts with ERK and dMP1 *in vivo*. We have previously shown that ERK and dMP1 co-immunoprecipitate in a total protein extract [3]. In order to determine the subcellular localization of this dMP1/ERK complex, we carried out co-immunoprecipitation experiments using cytoplasmic or nuclear extracts of S2 cells expressing dMP1 and ERK tagged proteins. Surprisingly, dMP1-Myc co-immunoprecipitated with ERK-FLAG in the nuclear extract only (Figure 7A). Corto-Myc and ERK-FLAG also co-immunoprecipitated in the nuclear extract only (Figure 7B). In this last experiment, all Corto isoforms were co-immunoprecipitated with ERK. In both cases, the lack of co-immunoprecipitation in cytoplasmic extracts was confirmed by using two different protocols to prepare nuclear and cytoplasmic extracts (see the Methods section). The co-immunoprecipitation observed between Corto and ERK fusion proteins was confirmed with endogenous proteins from a total embryonic extract using anti-dP-ERK antibody (Figure 7C). Furthermore, this experiment showed that Corto was able to interact with dP-ERK. Lastly, we observed no co-immunoprecipitation between Corto-FLAG and dMP1-Myc whether in total, cytoplasmic or nuclear extracts (data not shown). Labile protein interactions can be stabilized by cross-linking before performing cell extracts, although such treatment does not allow separating cytoplasmic and nuclear extracts. When using a total extract from cross-linked cells, we could detect co-immunoprecipitation between Corto-FLAG and dMP1-Myc (Figure 7D). This observation was consistent with our GST pull-down and peptide pull-down assays showing that the interaction between dMP1 and Corto was not direct but was probably mediated by other proteins. Altogether, our co-immunoprecipitation results suggest that a complex containing Corto, ERK and dMP1 might exist in the nucleus only. The core protein of this complex should be ERK, since it interacts directly with both Corto and dMP1.

### ERK and dMP1 bind polytene chromosomes where they partially co-localize with Corto

**Figure 8:**
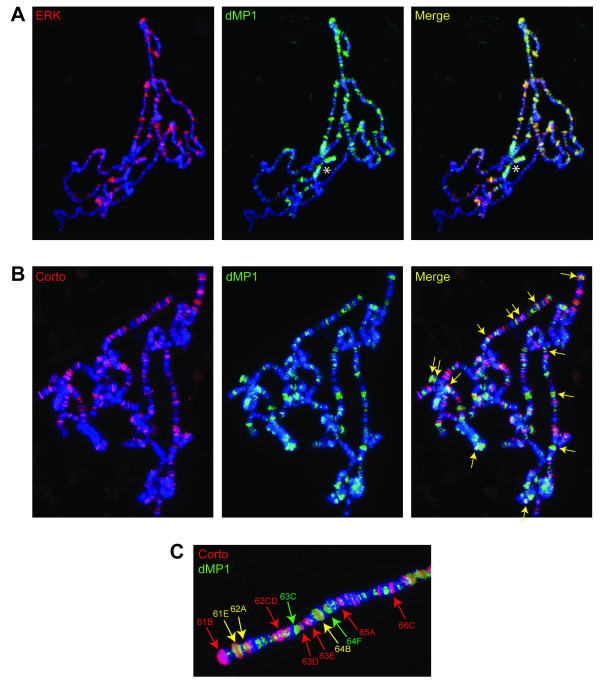
**ERK and dMP1 bind polytene chromosomes where they partially co-localize with Corto**. (A): Immunostaining of polytene chromosomes using rabbit anti-ERK (C-16) (left), and guinea-pig anti-dMP1 (center). DNA was stained with DAPI. ERK and dMP1 co-localize on all sites (right). The staining observed with dMP1 antibodies on centromeric heterochromatin and chromosome four (asterisk) is non-specific, since the same staining was observed with the pre-immune serum (data not shown). (B): Immunostaining of polytene chromosomes with rabbit anti-Corto antibodies (left) and guinea-pig anti-dMP1 antibodies (center). Corto and dMP1 co-localize on a number of sites (right, arrows). (C): Magnification of chromosome 3L extremity showing that Corto and dMP1 co-localize on several sites (yellow arrows).

To further analyze the relationship between Corto, dMP1 and ERK, and since Corto has been shown to bind polytene chromosomes [26], we analyzed the binding of ERK and dMP1 onto polytene chromosomes. We observed that ERK and dMP1 bound polytene chromosomes on many sites where they completely co-localized (Figure 8A). Furthermore, Corto and dMP1 co-localized on several sites (Figure 8B-C). These results suggest that a dMP1/ERK/Corto complex might regulate targets directly on chromatin. Since the co-localization of Corto with dMP1 is not complete, Corto appears to be an optional partner of dMP1/ERK on chromatin. Corto association may require additional factors or may be controlled by signaling events.

Previously, other scaffold proteins have been reported to bind chromatin. This is the case of the scaffold protein Ste5p in the pheromone pathway of yeast which interacts with the MAPKs Fus3p and Kss1p and occupies the same mating-type genes. Ste5p has then been suggested to function as an adaptor for protein-protein interactions both at the plasma membrane and in the nucleus [10]. In mammals, the scaffold protein β-arrestin, localized both in the cytoplasm and in the nucleus, is also recruited to target promoters under opioid receptor stimulation thus enhancing gene transcription [52]. The scaffold protein dMP1 could serve as an adaptor to connect ERK with other partners directly on chromatin. It could also allow ERK to form dimers, as mammalian scaffold proteins have been shown to be essential to connect ERK dimers to cytoplasmic targets [53]. It would therefore be interesting to know if ERK is monomeric or dimeric when bound to chromatin.

## Conclusions

We show here that the ETP *corto*, *rl *and *dMP1 *interact during wing tissue differentiation in *Drosophila*. Corto, ERK and dMP1 form a complex exclusively in the nucleus. In addition, these proteins bind polytene chromosomes where they partially co-localize, suggesting that the Corto-ERK-dMP1 complex might regulate vein and/or intervein gene expression directly on chromatin. Future experiments will be needed to test whether this complex, *via *the ETP Corto, participates in the recruitment of TrxG complexes on target genes in response to ERK signaling.

## Methods

### *Drosophila *strains and genetic crosses

Flies were raised on standard yeast-cornmeal medium at 25°C. *w^1118 ^*was used as control strain. The *corto^L1 ^*(EMS-induced allele), *corto^07128b^*, *bs^EY23316^*, *rho^EP3704 ^*(P-insertion alleles) lines were from the Bloomington Stock Center. The *corto^420 ^*line results from imprecise P-element excision [24,25]. The transgenic lines *UAS::corto *[24] (transgene on the third chromosome), *UAS::rolled *(transgene on the X chromosome) and *UAS::rl^Sem ^*[54] (transgene on the third chromosome) were gifts from Dr. R. Rosset (*UAS::corto*) and Dr. K. Moses (*UAS::rl *and *UAS::rl^Sem^*). The *UAS::dsMP1 *line allowing *dMP1 *down-regulation by RNA interference (transgene on the second chromosome) was described previously [3]. Lines containing a transgene with *UAS *sequences were crossed with the *hs::Gal4*, *scalloped::Gal4 *(*sd::Gal4*, [55]) and *Beadex::Gal4 *(*Bx::Gal4*, [56]) drivers. All crosses were performed at 25°C, except those of *UAS::rl^Sem ^*with *sd::Gal4 *that were performed at 18°C to decrease Gal4 activity and therefore lower transgene expression. To perform staged *corto *expression with the *hs::Gal4 *driver, Gal4 was induced by 20 minute heat-shocks applied at various moments during larval and pupal development.

### *In situ *hybridization experiments on pupal wings

White pupae were collected and maintained for 30 h at 25°C. After puparium dissection, pupae were fixed in 8% formaldehyde for 12 h at 4°C. *In situ *hybridization with *blistered *(EST SD23611) and *rhomboïd *(EST RE59529) DIG-UTP labeled RNA probes was performed according to standard protocols [57].

### Q-RT PCR experiments

Total RNA were extracted from 20 third instar larval discs of each genotype using the PureLink RNA Microkit (Invitrogen) according to the manufacturer's instructions. 1 μg of RNA was reverse-transcribed with the SuperScript^® ^VILO™ cDNA Synthesis Kit (Invitrogen). Q-RT PCR experiments were carried out on a Light Cycler 480 (Roche Diagnostics) with the Maxima SybrGreen mix (Fermentas). The primers used were: *cortoF *(5'-TGGCCACAGTTCCTAGCATT-3') and *cortoR *(5'-GCATGGGATTGGTGTCAGG-3'); *rp49F *(5'-CCGCTTCAAGGGACAGTATC-3') and *rp49R *(5'-GACAATCTCCTTGCGCTTC-3'); *spt6F *(5-'CGGAGGAGCTCTTCGATATG-3') and *spt6R *(5'-GACAGCTCTGGGAAGTCGTC-3'). A standard curve of amplification efficiency for each set of primers was generated with a serial dilution of cDNA. *rp49 *or *spt6 *levels were used for normalization according to the standard curve method. Three independent experiments were performed.

### Plasmids and S2 cell transfection

The *corto*, *dMP1*, *rl *and *rl^Sem ^*cDNAs were cloned into Gateway^® ^*Drosophila *vectors allowing expression of the fusion proteins under control of the *actin5C *promoter, as previously described [3]. The *rl^Sem ^*sequence was obtained by *in vitro *mutagenesis using the QuickChange^® ^Site Directed Mutagenesis kit (Stratagene) according to the manufacturer's instructions. This gain-of-function mutation is a G to A transition resulting in a D to N substitution at position 334 [28]. pMT-Ras^V12 ^(a gift from Dr. A. Nagel) allowed transient expression of the constitutively active form of Ras, Ras^V12^, under the control of the heavy metal inducible promoter *metallothionein *[58]. Ras^V12 ^was induced by treating transfected cells for 24 h with CuSO_4 _0.5 mM. For transfection, S2 cells were cultivated at 25°C in Schneider medium with or without 10% fetal calf serum as indicated. 5.10^6 ^cells were transfected with 2 μg of DNA using Effecten^® ^transfection reagent (Qiagen) according to the manufacturer's instructions (1/10 DNA-Effecten^® ^ratio). Cells were collected 48 h (ERK pathway activation) or 72 h (immunoprecipitation) after transfection.

### Protein extracts and phosphatase treatment

Embryos and S2 cell total extracts were prepared by sonication in RIPA buffer [50 mM Tris-HCl pH7.5, 150 mM NaCl, 25 mM NaVO_4_, 25 mM NaF, 0.1% SDS, 0.5% NP40, complete protease inhibitors (Roche)]. Cytoplasmic extracts were prepared either with NE-PER Nuclear and Cytoplasmic Extraction Reagents (Pierce) according to the manufacturer's instructions or by homogenization using a Dounce potter in low salt buffer as described in [59]. When analyzed by immunoprecipitation, these two kinds of cytoplasmic extracts gave the same results. Nuclear extracts were obtained by sonication of the nuclear pellet in RIPA buffer. Phosphatase treatments of S2 cell extracts prepared without NaVO_4 _and NaF phosphatase inhibitors were performed using Lambda Protein Phosphatase (Upstate) for a 10 minute incubation time at 37°C.

### Western blot analysis and antibodies

Cell lysates or immunoprecipitated proteins were resolved on 8% SDS-PAGE Anderson gels [60] when separating different isoforms of Corto, or on 12% or 15% classical SDS-PAGE depending on the molecular weight of proteins. Western blot experiments were performed according to standard protocols. Antibodies used were monoclonal anti-FLAG (F3165, Sigma) or anti-Myc (sc-40, Santa Cruz Biotechnology) antibodies for fusion proteins, anti-β- tubulin (E7) and anti-lamin (ADL67.10) antibodies (Developmental Studies Hybridoma Bank) for control of cytoplasmic and nuclear fractions, rat anti-Corto antibodies [26], monoclonal phosphoserine/threonine/tyrosine antibody (MA1-38450, Pierce), rabbit anti-ERK antibodies (C-16, Santa-Cruz Biotechnology) or monoclonal anti-dP-ERK E10 antibody (9106, Cell Signaling). Anti-HA antibody (H3663, Sigma) was used as a negative control in immunoprecipitation experiments.

### Protein-protein interactions

*In vitro *transcription-translation and GST pull-down assays were performed as previously described [26] using GST-Corto fusion proteins [61] and GST-dMP1 fusion protein [3].

For co-immunoprecipitation experiments, 500 μg of protein extracts (total, cytoplasmic or nuclear) were immunoprecipitated either with monoclonal anti-FLAG antibody, anti-Myc 9E10 antibody or anti-HA antibody using magnetic protein G-agarose beads (Ademtech). To co-immunoprecipitate Corto and dMP1, proteins were cross-linked before extraction by treating cells with 1% formaldehyde for 10 minutes followed by neutralization with 0.13 M glycine. To co-immunoprecipitate Corto and dP-ERK in embryonic extracts, monoclonal anti-dP-ERK E10 antibody was covalently bound onto protein G-agarose beads using standard protocols.

Two-hybrid experiments were performed as previously described [26], using leucine and X-Gal tests. Both tests gave the same result, and only the leucine test is shown. The full length dMP1 protein was fused with the B42 activation domain (B42AD). The NH_2_-terminal half of Corto was fused with the LexA DNA binding domain. B42AD and LexA were used as negative controls. B42/SV40 large T-antigen (B42ADT) and LexA/p53 (LexA53) fusion proteins were used as positive controls.

### Immunolocalization on polytene chromosomes

Co-immunostaining of *w^1118 ^*polytene chromosomes was performed as previously described [26] using rabbit anti-ERK (1:20) (C-16; Santa-Cruz Biotechnology), guinea-pig anti-dMP1 (1:20) [3] and rabbit anti-Corto (1:20) [26] as primary antibodies. Secondary antibodies (Alexa Fluor^® ^594 goat anti-rabbit IgG and Alexa Fluor^® ^488 goat anti-guinea-pig IgG, Molecular Probes) were used at a 1:1000 dilution.

## Authors' contributions

EM-V. conceived and performed all the genetics experiments and most of the biochemical experiments. JR and MD participated in biochemical experiments. JR performed quantitative PCR experiments. FP performed *in situ *hybridization experiments and polytene chromosome immunostainings. EM-V and FP wrote the paper. All the authors read and approved the final manuscript.
